# Metabolic profiles to predict long-term cancer and mortality: the use of latent class analysis

**DOI:** 10.1186/s12860-019-0210-7

**Published:** 2019-07-23

**Authors:** Aida Santaolalla, Hans Garmo, Anita Grigoriadis, Sundeep Ghuman, Niklas Hammar, Ingmar Jungner, Göran Walldius, Mats Lambe, Lars Holmberg, Mieke Van Hemelrijck

**Affiliations:** 1 School of Cancer & Pharmaceutical Sciences, Translational Oncology and Urology Research, King’s College London, London, UK; 2 Regional Oncologic Centre, Uppsala University, Uppsala, Sweden; 3 School of Cancer & Pharmaceutical Sciences, Cancer Bioinformatics, Breast Cancer Now, King’s College London, London, UK; 4 Guy’s and St Thomas, NHS Foundation Trust, London, UK; 5 Department of Epidemiology, Institute of Environmental Medicine, Karolinska Institutet, Stockholm, Sweden; 6 Department of Medicine, Clinical Epidemiological Unit, Karolinska Institutet and CALAB Research, Stockholm, Sweden; 7 Unit of Cardiovascular Epidemiology, Institute of Environmental Medicine, Karolinska Institutet, Stockholm, Sweden; 8 Department of Medical Epidemiology and Biostatistics, Karolinska Institutet, Stockholm, Sweden

**Keywords:** Risk stratification, Biomarkers, Metabolic profiles, Latent class analysis, Disease susceptibility, Cancer epidemiology

## Abstract

**Background:**

Metabolites are genetically and environmentally determined. Consequently, they can be used to characterize environmental exposures and reveal biochemical mechanisms that link exposure to disease. To explore disease susceptibility and improve population risk stratification, we aimed to identify metabolic profiles linked to carcinogenesis and mortality and their intrinsic associations by characterizing subgroups of individuals based on serum biomarker measurements. We included 13,615 participants from the Swedish Apolipoprotein MOrtality RISk Study who had measurements for 19 biomarkers representative of central metabolic pathways. Latent Class Analysis (LCA) was applied to characterise individuals based on their biomarker values (according to medical cut-offs), which were then examined as predictors of cancer and death using multivariable Cox proportional hazards models.

**Results:**

LCA identified four metabolic profiles within the population: (1) normal values for all markers (63% of population); (2) abnormal values for lipids (22%); (3) abnormal values for liver functioning (9%); (4) abnormal values for iron and inflammation metabolism (6%). All metabolic profiles (classes 2–4) increased risk of cancer and mortality, compared to class 1 (e.g. HR for overall death was 1.26 (95% CI: 1.16–1.37), 1.67 (95% CI: 1.47–1.90), and 1.21 (95% CI: 1.05–1.41) for class 2, 3, and 4, respectively).

**Conclusion:**

We present an innovative approach to risk stratify a well-defined population based on LCA metabolic-defined subgroups for cancer and mortality. Our results indicate that standard of care baseline serum markers, when assembled into meaningful metabolic profiles, could help assess long term risk of disease and provide insight in disease susceptibility and etiology.

**Electronic supplementary material:**

The online version of this article (10.1186/s12860-019-0210-7) contains supplementary material, which is available to authorized users.

## Background

Cancer is a multi-pathway disease, assembled as a heterogeneous and hierarchically organized system, and still one of the major causes of death worldwide – with an increasing burden given the aging population [1–3]. Cancer data has grown exponentially in the last decade with new advanced technologies resulting in highly diverse, mixed data types and huge volumes of information (e.g.: 542045 is the number of publications retrieved in PubMed when searching the terms ‘cancer’ AND ‘data’ on August 2017). Due to the nature of this emerged “Big Cancer Data” and the demand for high-sensitive and high-specific biomarkers, there is a need for significant sample sizes and advanced mathematical and statistical models [4, 5] capable of extracting relevant clinical and biological information [6, 7]. These more systematic-based approaches, replacing single biomarker analyses by multiple profiling testing, may provide new avenues for biomarker development in cancer diagnosis and management [8, 9]. Recent studies have adopted these integrative approaches assessing multiple serum markers simultaneously for cancer diagnosis [10–13]. Furthermore, the concept of the exposome has been introduced into the field of cancer epidemiology [14]. It refers to every non-genetic exposure to which an individual is subjected from conception to death [14, 15] . Specifically, metabolites, part of the internal exposome, are both genetically and environmentally determined and can consequently be used to characterize environmental exposures and reveal biochemical mechanisms that link exposure to disease [15–18]. Hence, the internal distribution of metabolites and their interactions might help unravelling cancer susceptibility in a population.

With the overall goal of identifying statistical methods to stratify individuals based on their underlying risk of developing cancer and risk of increasing mortality, we conducted a data driven approach utilizing standard serum markers available from routine health check-ups to study susceptibility to cancer and death in a well-defined cohort of 13,615 participants from the AMORIS study (Apolipoprotein MOrtality RISk) [19, 20]. More specifically, the study was set out to explore population heterogeneity and cancer susceptibility by investigating serum metabolic profiles using latent class analysis (LCA). This data reduction method clusters covariates based on models of data distribution probabilities. It allows for evaluation of clusters of biomarkers linked to carcinogenesis and their intrinsic associations, which ultimately helps us assess their possible role in predicting long-term cancer and mortality.

## Results

### Characteristics of the study population

A total of 1,956 individuals (14.37%) developed cancer after at least 3 years of follow-up, including 655 breast and genito-urinary cancers, 330 cases of digestive cancer, 133 cases of respiratory cancers and 129 lymphatic and hematopoietic cancers during a mean follow-up time for cancer of 16.6 years, median follow-up time in the cohort of 17.22 years with a minimum of 3.01 years and a maximum of 24.77. Three thousand one hundred fifty-eight participants (23.20%) died during a mean follow-up of 17.3 years, comprising 706 cancer-specific deaths. Study population characteristics by cancer status is illustrated in Table 1.

**Table 1:** Characteristics of the study population by cancer status defined at the end of the follow up period. All the serum markers are dichotomized using standard clinical cut-offs

	Total *N* = 13,615 (100%)	No Cancer *N* = 11,659 (85.63%)	Cancer *N* = 1,956 (14.37%)
Age (years)			
Mean (SD)	51.91 (14.80)	50.86 (15.00)	58.14 (11.75)
Under 40	2951 (21.67)	2841 (24.37)	110 (5.62)
40–50	3550 (26.07)	3148 (27.00)	402 (20.55)
50–60	3065 (22.51)	2491 (21.37)	574 (29.35)
Above 60	4049 (29.74)	3179 (27.27)	870 (44.48)
Sex			
Female	7588 (55.73)	6636 (56.92)	952 (48.67)
Male	6027 (44.27)	5023 (43.08)	1004 (51.33)
Socio-economics Status			
High	6493 (47.69)	5416 (46.45)	1077 (55.06)
Low	5007 (36.78)	4368 (37.46)	639 (32.67)
Not employed or missing	2115 (15.53)	1875 (16.08)	240 (12.27)
Educational Status			
High	4313 (33.42)	3688 (33.40)	625 (33.57)
Middle	5495 (42.58)	4725 (42.79)	770 (41.35)
Low	3097 (24.00)	2630 (23.82)	467 (25.08)
Missing ^b^	710 (5.21)	616 (5.28)	94 (4.80)
CCI			
0	12258 (90.03)	10520 (90.23)	1738 (88.85)
1	963 (7.07)	807 (6.92)	156 (7.98)
2	221 (1.62)	188 (1.61)	33 (1.69)
**3**+	173 (1.27)	144 (1.24)	29 (1.48)
Total Cholesterol (mmol/L)			
Mean(SD)	5.82 (1.17)	5.79 (1.18)	6.00 (1.13)
< 6.50	9774 (71.79)	8453 (72.50)	1321 (67.54)
≥ **6.50**	3841 (28.21)	3206 (27.50)	635 (32.46)
Triglycerides (mmol/L)			
Mean(SD)	1.44 (1.00)	1.43 (1.00)	1.48 (0.93)
< 1.71	10128 (74.39)	8716 (74.76)	1412 (72.19)
**≥ 1.71**	3487 (25.61)	2943 (25.24)	544 (27.81)
Apolipoprotein A-1 (g/L)			
Mean(SD)	1.44 (0.23)	1.44 (0.23)	1.43 (0.23)
**< 1.05**	328 (2.41)	278 (2.38)	50 (2.56)
≥ 1.05	13287 (97.59)	11381 (97.62)	1906 (97.44)
Apolipoprotein B (g/L)			
Mean(SD)	1.22 (0.35)	1.22 (0.35)	1.29 (0.34)
< 1.50	10902 (80.07)	9431 (80.89)	1471 (75.20)
≥ **1.50**	2713 (19.93)	2228 (19.11)	485 (24.80)
HDL Cholesterol (mmol/L)			
Mean(SD)	1.54 (0.43)	1.54 (0.43)	1.52 (0.43)
**< 1.03**	1457 (10.70)	1231 (10.56)	226 (11.55)
≥ 1.03	12158 (89.30)	10428 (89.44)	1730 (88.45)
LDL Cholesterol (mmol/L)			
Mean(SD)	3.64 (1.06)	3.61 (1.06)	3.82 (1.04)
< 4.10	9345 (68.64)	8128 (69.71)	1217 (62.22)
≥ **4.10**	4270 (31.36)	3531 (30.29)	739 (37.78)
Glucose (mmol/L)			
Mean(SD)	5.22 (1.53)	5.21 (1.53)	5.30 (1.53)
< 6.11	12223 (89.78)	10488 (89.96)	1735 (88.70)
≥ **6.11**	1392 (10.22)	1171 (10.04)	221 (11.30)
Fructosamine (mmol/L)			
Mean(SD)	2.09 (0.27)	2.08 (0.27)	2.10 (0.25)
< 2.6	13184 (96.83)	11291 (96.84)	1893 (96.78)
≥ **2.6**	431 (3.17)	368 (3.16)	63 (3.22)
GGT (IU/L) ^d^			
Mean(SD)	33.21 (48.12)	32.74 (48.09)	36.03 (48.21)
Normal (< 18)	5511 (40.48)	4827 (41.40)	684 (34.97)
**Normal high (18–36)**	4983 (36.60)	4236 (36.33)	747 (38.19)
**Elevated (36–72)**	2098 (15.41)	1750 (15.01)	348 (17.79)
**Highly elevated (> 72)**	1023 (7.51)	846 (7.26)	177 (9.05)
AST (IU/L)			
Mean(SD)	22.84 (19.23)	22.70 (19.60)	23.64 (16.88)
< 45	13155 (96.62)	11271 (96.67)	1884 (96.32)
**≥ 45**	460 (3.38)	388 (3.33)	72 (3.68)
ALT (IU/L)			
Mean(SD)	29.02 (34.35)	28.95 (35.73)	29.41 (24.54)
< 50	12296 (90.31)	10546 (90.45)	1750 (89.47)
≥ **50**	1319 (9.69)	1113 (9.55)	206 (10.53)
Albumin (g/L)			
Mean(SD)	43.05 (2.82)	43.13 (2.83)	42.58 (2.72)
**< 35**	28 (0.21)	23 (0.20)	5 (0.26)
> 35	13587 (99.79)	11636 (99.80)	1951 (99.74)
Leukocytes (10^9^ cells/L)			
Mean(SD)	6.52 (1.97)	6.49 (1.96)	6.65 (2.01)
< 10	12956 (95.16)	11106 (95.26)	1850 (94.58)
≥ **10**	659 (4.84)	553 (4.74)	106 (5.42)
C-Reactive Protein (mg/L)			
Mean(SD)	5.86 (15.14)	5.82 (14.25)	6.16 (19.58)
< 10	11858 (87.1)	10193 (87.43)	1665 (85.12)
**10–15**	1196 (8.78)	993 (8.52)	203 (10.38)
**15–25**	265 (1.95)	223 (1.91)	42 (2.15)
**25–50**	200 (1.47)	167 (1.43)	33 (1.69)
**> 50**	96 (0.71)	223 (0.71)	13 (0.66)
Iron (μmol/L) ^d^			
Mean(SD)	18.13 (5.80)	18.13 (5.83)	18.11 (5.59)
**Low**	636 (4.67)	540 (4.63)	96 (4.91)
Normal	12512 (91.90)	10715 (91.90)	1797 (91.87)
**High**	467 (3.43)	404 (3.47)	63 (3.22)
TIBC (mg/dL) ^d^			
Mean(SD)	0.39 (0.11)	0.31 (0.11)	0.31 (0.10)
**Low**	4067 (29.87)	3494 (29.97)	573 (29.29)
Normal	6650 (48.84)	5683 (48.74)	967 (49.44)
**High**	2898 (21.29)	2482 (21.29)	416 (21.27)
Creatinine (μmol/L) ^d^			
Mean(SD)	79.65 (16.16)	79.38 (16.37)	81.26 (14.74)
Low	40 (0.29)	31 (0.27)	9 (0.46)
Normal	12088 (88.78)	10392 (89.13)	1696 (86.71)
**High**	1487 (10.92)	1236 (10.60)	251 (12.83)
Phosphate (mmol/L) ^d^			
Mean(SD)	1.07 (0.17)	1.07 (0.17)	1.05 (0.17)
Low	95 (0.70)	76 (0.65)	19 (0.97)
Normal	12796 (93.98)	10948 (93.90)	1848 (94.48)
**High**	724 (5.32)	635 (5.45)	89 (4.55)
Calcium (mmol/L) ^d^			
Mean(SD)	2.38 (0.09)	2.38 (0.09)	2.38 (0.10)
Low	191 (1.40)	167 (1.43)	24 (1.23)
Normal	13195 (96.92)	11300 (96.92)	1895 (96.88)
**High**	229 (1.68)	192 (1.65)	37 (1.89)
Log (triglycerides/HDL) c			
mean(SD)	(−)0.19 (0.81)	(−)0.20 (0.82)	(−)0.14 (0.80)
< 0.5	11197 (82.24)	9618 (82.49)	1579 (80.73)
**≥ 0.5**	2418 (17.76)	2041 (17.51)	377 (19.27)
ApoB/ApoA-I c			
mean(SD)	0.87 (0.29)	0.87 (0.29)	0.92 (0.30)
< 1.00	9584 (70.39)	8347 (71.59)	1237 (63.24)
**≥ 1.00**	4031 (29.61)	3312 (28.41)	719 (36.76)
Life Status			
Alive	10457 (76.80)	9385 (80.50)	1072 (54.81)
Death	3158 (23.20)	2274 (19.50)	884 (45.19)
Cancer	1956 (14.90)	11659 (0.00)	1956 (100.00)

The following abbreviations have been used in Table 1: High Density Lipoprotein (HDL), Low Density Lipoprotein (LDL), Gamma-Glutamyl transferase (GGT), Alanine aminotransferase (ALT), Aspartate aminotransferase (AST) and Total iron binding capacity (TIBC).

^a^Clinically abnormal cut-off values are highlighted for each biomarker.

^b^The missing values are not included in the percentage of the Educational Status categories.

^c^Ratios are dimensionless.

^d^Clinical cut-offs

The following cut-offs criteria was applied:

**GGT reference interval:**

Low [GGT < 18 IU/L].

Normal high [18 IU/L ≥ GGT < 36 IU/L].

Elevated [36 IU/L ≥ GGT < 72 IU/L].

High elevated [GGT ≥ 72 IU/L].

**Iron reference interval:**

Men [Low ≤11, Normal = 11–31, High ≥31].

Women [Low ≤9, Normal = 9–30, High≥30].

**TIBC reference interval:**

Men [Low ≤0.257, Normal = 0.257–0.379, High ≥0.379].

Women [Low ≤0.246, Normal = 0.246–0.391, High ≥0.391].

**Creatinine reference interval:**

Men [Low ≤60, Normal = 60–100, High ≥100].

Women [Low ≤45, Norma l = 45–90, High ≥90].

**Phosphate reference interval:**

Men [Low ≤0.7, Normal = 0.7–1.4, High ≥1.4].

Women [Low ≤0.8, Normal = 0.8–1.4, High ≥1.4].

**Calcium reference interval per gender by age:**

Men

[Age < 40, Low ≤2.22, Normal = 2.22–2.60, High ≥2.60].

[Age 40–60, Low ≤2.20, Normal = 2.20–2.59, High ≥2.59]

[Age > 60, Low ≤2.19, Normal = 2.19–2.58, High ≥2.58]

Women

[Age < 40, Low ≤2.17, Normal = 2.17–2.56, High ≥2.56].

[Age 40–60, Low ≤2.19, Normal = 2.19–2.60, High ≥2.60]

[Age > 60, Low ≤2.21, Normal = 2.21–2.60, High ≥2.60]

Abnormal clinical cut-off values of the biomarkers are indicated in the table in boldface

### Latent class analysis characterizes the study population into four metabolic profiles

**Fig. 1:**
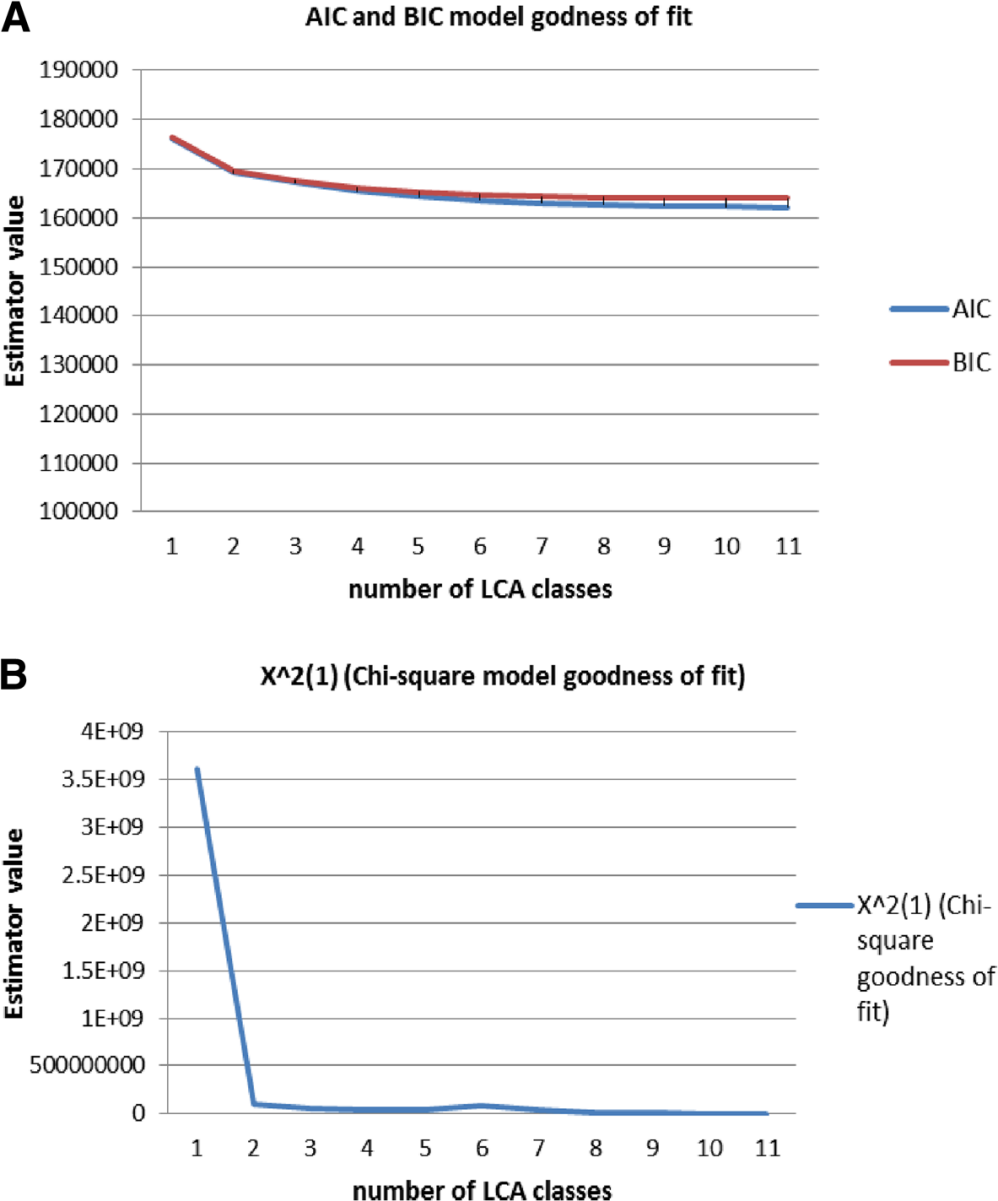
**a** Line-graph depicting the goodness of fit indicators AIC and BIC. The model that best fits the dataset comprehends of four latent classes as determined by the minimum value reached by AIC and BIC criterions before stabilization of the values. The criterion did not converge to a local maximum from class 12 onwards. **b** Line-graph depicting the goodness of fit indicators (X^2 (1) (Chi-square). The model that best fits the dataset comprehends of four latent classes as determined by the minimum value reached by Chi-square. The criterions did not converge to a local maximum from class 12 onwards

LCA was executed using the dichotomized values of the biomarkers to facilitate the biological interpretation of the results. The Chi-squared distribution criterion for model selection indicated a best fit model comprehend of four LCA classes, while AIC and BIC stabilized at 4 classes (Fig. 1a, b) [21]. All the criterions did not converge to a local maximum from class 12 onwards. The class allocation of the observations (individuals), the class conditional probability of each biomarker and the latent mixing proportions were obtained when running poLCA package in R statistical language.

**Fig. 2:**
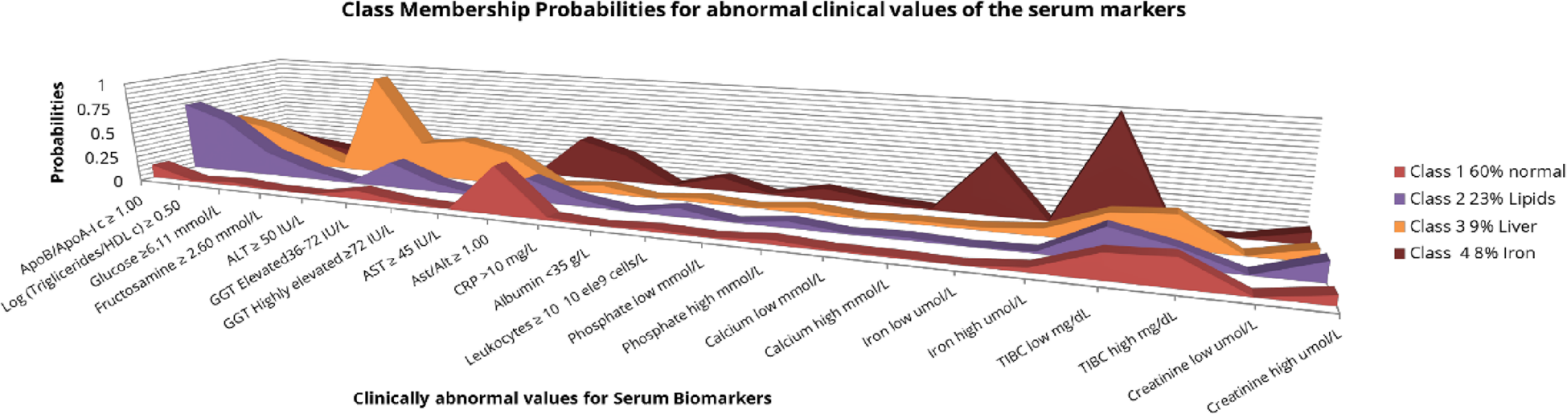
Class Membership Probabilities for abnormal clinical values of the serum markers for the four LCA – derived metabolic classes. The four different biomarker profiles are represented in the graph

Table 2 and Fig. 2 outline the LCA-derived classes with the estimated class population proportions, the class conditional probabilities of belonging to each latent class for each of the biomarkers and the biological interpretation of the LCA-derived classes. The four mutually exclusive classes characterize the population in metabolic profiles based on class conditional probabilities: (1) those with probabilities for all abnormal values of the markers under 0.3; therefore, considered the normal class (63% of population); (2) those with abnormal values for lipid markers (22%); (3) those with abnormal values for liver function markers (9%); (4) those with abnormal values for iron and inflammation metabolism (6%).

**Table 2:** Predicted class memberships of the clinically abnormal biomarkers cut-off values for the 4 latent classes model. Estimated class population shares for the four different LCA classes

LCA-derived Classes	Class 1	Class 2	Class 3	Class 4
% on the population	63%	22%	9%	6%
Biological interpretation	Normal	Lipids	Liver	Iron/ Inflammation
ApoB/ApoA-I ≥ 1.00 b	0.1320	**0.6840**	0.4519	0.2480
Log (Triglycerides/HDL) ≥ 0.50 b	0.0126	**0.5436**	0.3852	0.1421
Glucose ≥6.11 mmol/L	0.0342	0.2401	0.2174	0.0919
Fructosamine ≥2.60 mmol/L	0.0039	0.0967	0.0555	0.0280
ALT ≥50 IU/L	0.0051	0.0107	**1.0000**	0.0291
GGT Elevated36–72 IU/L	0.0848	0.2532	**0.3521**	0.1732
GGT Highly elevated ≥72 IU/L	0.0240	0.0843	**0.4098**	0.0619
AST ≥ 45 IU/L	0.0052	0.0045	**0.3168**	0.0180
CRP > 10 mg/L	0.0282	0.0715	0.0771	**0.2740**
Albumin < 35 g/L	0.0007	0.0022	0.0024	0.0114
Leukocytes ≥10^9^ cells/L	0.0265	0.0786	0.0438	0.1344
Iron low μmol/L	0.0001	0.0040	0.0281	**0.5527**
Iron high μmol /L	0.0404	0.0155	0.0712	0.0000
TIBC low mg/dL	0.2201	0.2807	0.2622	**1.0000**
TIBC high mg/dL	0.2438	0.1707	0.2984	0.0000
Creatinine low μmol /L	0.0022	0.0037	0.0041	0.0051
Creatinine high μmol /L	0.0822	0.1765	0.1166	0.1116
Phosphate low mmol/L	0.0078	0.0041	0.0063	0.0098
Phosphate high mmol/L	0.0425	0.0611	0.0544	0.1110
Calcium low mmol/L	0.0124	0.0092	0.0099	0.0458
Calcium high mmol/L	0.0121	0.0253	0.0299	0.0135

^a^High probabilities of the biomarkers to belong to a class are highlighted.

^b^Ratios are dimensionless.

Higher proportions of the abnormal biomarkers belonging to a class is indicated in the table in boldface

A validation of the characterization of the population performed with the Latent class methodology is outlined in Additional file 1: Table S3. The baseline clinical characteristics of the individuals by LCA-derived metabolic classes (Additional file 1: Table S3) replicate the results displayed in Table 2 for the class conditional probabilities.

### LCA derived metabolic profiles as cancer and mortality predictors

We then investigated the prediction capabilities of the four LCA-derived metabolic profiles to estimate overall cancer risk, specific cancer types risk, cancer mortality and overall mortality, assigning the reference level to the healthy metabolic profile Class 1 (Tables 3 and 4).

**Table 3:** Hazard ratios and 95% confidence interval for the association of LCA-derived metabolic classes and overall cancer risk and cancer specific risk

	Hazard Ratios (95% CI) ^a^	Hazard Ratios (95% CI) ^b^
Cancer Risk: All cancer types		
Number of events	1956	1956
1 - Normal class	1.00 (ref)	1.00 (ref)
2 - Lipids	1.09 (0.98–1.22)	1.05 (0.94–1.17)
3 - Liver	1.28 (1.10–1.50)	1.28 (1.09–1.49)
4 – Inflammation & Iron	1.17 (0.97–1.41)	1.17 (0.97–1.41)
Cancer Risk: Buccal cavity and pharynx		
Number of events	34	34
1 - Normal class	1.00 (ref)	1.00 (ref)
2 - Lipids	1.79 (0.77–4.14)	1.70 (0.73–1.17)
3 - Liver	2.66 (0.96–7.35)	2.60 (0.94–7.16)
4 - Inflammation & Iron	3.94 (1.38–11.30)	3.77 (1.31–10.82)
Cancer Risk: Digestive organs and peritoneum		
Number of events	133	133
1 - Normal class	1.00 (ref)	1.00 (ref)
2 - Lipids	0.83 (0.62–1.11)	0.83 (0.62–1.11)
3 - Liver	2.12 (1.54–2.91)	2.12 (1.54–2.91)
4 - Inflammation & Iron	0.86 (0.51–1.46)	0.86 (0.51–1.46)
Cancer Risk: Respiratory system		
Number of events	133	133
1 - Normal class	1.00 (ref)	1.00 (ref)
2 - Lipids	1.40 (0.94–2.08)	1.32 (0.88–1.96)
3 - Liver	0.90 (0.44–1.82)	0.87 (0.43–1.77)
4 - Inflammation & Iron	1.48 (0.76–2.88)	1.46 (0.75–2.84)
Cancer Risk: Skin melanoma		
Number of events	205	205
1 - Normal class	1.00 (ref)	1.00 (ref)
2 - Lipids	0.78 (0.56–1.10)	0.78 (0.56–1.11)
3 - Liver	0.71 (0.40–1.26)	0.73 (0.41–1.31)
4 - Inflammation & Iron	0.70 (0.35–1.37)	0.70 (0.35–1.37)
Cancer Risk: Breast and genito-urinary organs		
Number of events	655	655
1 - Normal class	1.00 (ref)	1.00 (ref)
2 - Lipids	1.19 (0.99–1.42)	1.12 (0.94–1.33)
3 - Liver	1.04 (0.80–1.37)	1.04 (0.80–1.37)
4 - Inflammation & Iron	1.25 (0.91–1.71)	1.25 (0.91–1.71)
Cancer Risk: Brain & nervous system, Thyroids		
Number of events	34	34
1 - Normal class	1.00 (ref)	1.00 (ref)
2 - Lipids	1.01 (0.51–1.99)	0.96 (0.48–1.00)
3 - Liver	1.01 (0.38–2.67)	0.99 (0.38–2.59)
4 - Inflammation & Iron	0.92 (0.28–2.99)	0.91 (0.28–2.96)
Cancer Risk: Connective and endocrine tissue		
Number of events	56	56
1 - Normal class	1.00 (ref)	1.00 (ref)
2 - Lipids	0.65 (0.21–1.95)	0.64 (0.21–1.94)
3 - Liver	2.65 (1.00–7.02)	2.67 (1.01–7.07)
4 - Inflammation & Iron	3.00 (1.11–8.11)	2.96 (1.10–8.00)
Cancer Risk: Lymphatic and hematopoietic tissues: Hodgkin lymphoma, Non-H lymphoma, Leukemia and Myeloma		
Number of events	129	129
1 - Normal class	1.00 (ref)	1.00 (ref)
2 - Lipids	1.72 (1.15–2.56)	1.68 (1.12–2.51)
3 - Liver	1.65 (0.91–3.00)	1.68 (0.93–3.05)
4 - Inflammation & Iron	1.23 (0.56–2.68)	1.25 (0.57–2.73)

^a^Time scale adjusted for age, sex and CCI.

^b^Age scale adjusted for age, sex and CCI.

**Table 4:** Hazard ratios and 95% confidence interval for the association of LCA- derived metabolic classes and all causes death and Cancer death

	Hazard Ratios (95% CI) ^a^	Hazard Ratios (95% CI) ^b^
All causes death		
Number of events	3158	3158
1 - Normal class	1.00 (ref)	1.00 (ref)
2 - Lipids	1.26 (1.16–1.37)	1.29 (1.19–1.40)
3 - Liver	1.67 (1.47–1.90)	1.70 (1.49–1.93)
4 - Inflammation & Iron	1.21 (1.05–1.41)	1.20 (1.04–1.40)
Cancer death		
Number of events	706	706
1 - Normal class	1.00 (ref)	1.00 (ref)
2 - Lipids	1.22 (1.02–1.45)	1.20 (1.01–1.42)
3 - Liver	1.44 (1.11–1.86)	1.46 (1.13–1.90)
4 - Inflammation & Iron	0.93 (0.66–1.32)	0.93 (0.66–1.32)

^a^Time scale adjusted for age, sex and CCI.

^b^Age scale adjusted for age, sex and CCI.

All metabolic profiles increased risk of cancer and mortality compared to Class 1. For instance, individuals in Class 3 (abnormal liver function profile) had a higher risk of overall cancer (HR: 1.28 (95% CI: 1.10–1.50)), but also a worse cancer-specific survival and overall survival as compared to those in Class 1 (Tables 3 and 4). Class 2 (abnormal lipid profile) and Class 4 (abnormal iron markers and inflammatory) were positively associated with overall death, while Class 2 was also associated with cancer–specific death. The results were consistent for both time-scales (Tables 3 and 4).

When assessing the risk of specific cancer types, several patterns occurred (Tables 3 and 4). Individuals in Class 2 (abnormal lipid markers) presented a higher risk of lymphatic and hematopoietic tissue cancer (HR: 1.72 (95% CI: 1.15–2.56)). There was a greater risk of digestive cancers in individuals in Class 3 (abnormal values of liver enzymes) (HR: 2.12 (95% CI: 1.54–2.91)), while individuals in Class 4 (abnormal iron markers and inflammation) were exposed to a higher risk of buccal and oral system cancers in comparison with the individuals in Class 1 (HR: 3.94 (95% CI 1.38–11.30)) (Table 3).

Moreover, the connective tissue and endocrine glands cancer risk was higher in individuals grouped in liver metabolic profile (HR: 2.65 (95% CI: 1.00–7.02) and in participants belonging to the iron markers and inflammation (HR: 3.00 (95% CI: 1.11–8.11)). Similar associations were observed when using the age scale for the multivariable cox proportional hazard regression model (Tables 3 and 4).

## Discussion

We demonstrated that standard of care baseline serum markers when assembled into meaningful metabolic profiles can help stratify the population for cancer risk, cancer mortality and overall mortality. More specifically, we observed that abnormal values for markers of the lipid metabolism, liver function and inflammatory and iron metabolism distinguish participants into metabolic profiles, which are predictive of long term cancer risk and/or mortality.

### Metabolic profiles

Among the biological pathways addressed in our LCA, abnormalities in the lipid metabolism were the most common. Hyperlipidemia was present in about a quarter of the study population explaining the largest abnormal metabolic profile. The weight of the lipid profile in the analysis was consistent with the reported global prevalence of hypercholesterolemia among adults (37% for males and 40% for females) as reported in the Global Health Observatory in 2008 estimates by the World Health Organization (WHO) and the results from the Swedish population in the WHO MONICA project [22]. Dyslipidemias are associated with higher risk of CVD and other chronic diseases such as cancer, as also observed in our study [23]. Liver dysfunction, iron deficiency and altered inflammatory markers profiles also distinguished important subgroups in our study population. About 9% of our population had abnormal values for markers of liver functioning (GGT, AST and ALT), which is similar to the results obtained in a population-based survey in the United States that estimated abnormal alanine aminotransferase (ALT) was present in 9% of respondents in absence of viral hepatitis C or excessive alcohol consumption [24]. Moreover, these enzymes are known to be linked to cancer because of their role in preserving the intracellular homeostasis of the oxidative stress [25–27], which is concordant with the results of these analyses. The iron profile and inflammatory markers clustered 6% of individuals in the study, which was predominantly driven by low levels of serum iron and TIBC, as well as high levels of CRP and leukocytes. This could potentially point towards anemia of inflammation, a chronic inflammation presenting low iron values, that occurs because the iron deficiency provides the body with infection resistance, which demonstrates the tightly connection between the inflammatory response and the iron and its homeostasis [28]. This condition has been reported in more than 30% of cancer patients at time of diagnosis.

### Metabolic profiles as a risk factor for long term cancer and mortality

**Fig. 3:**
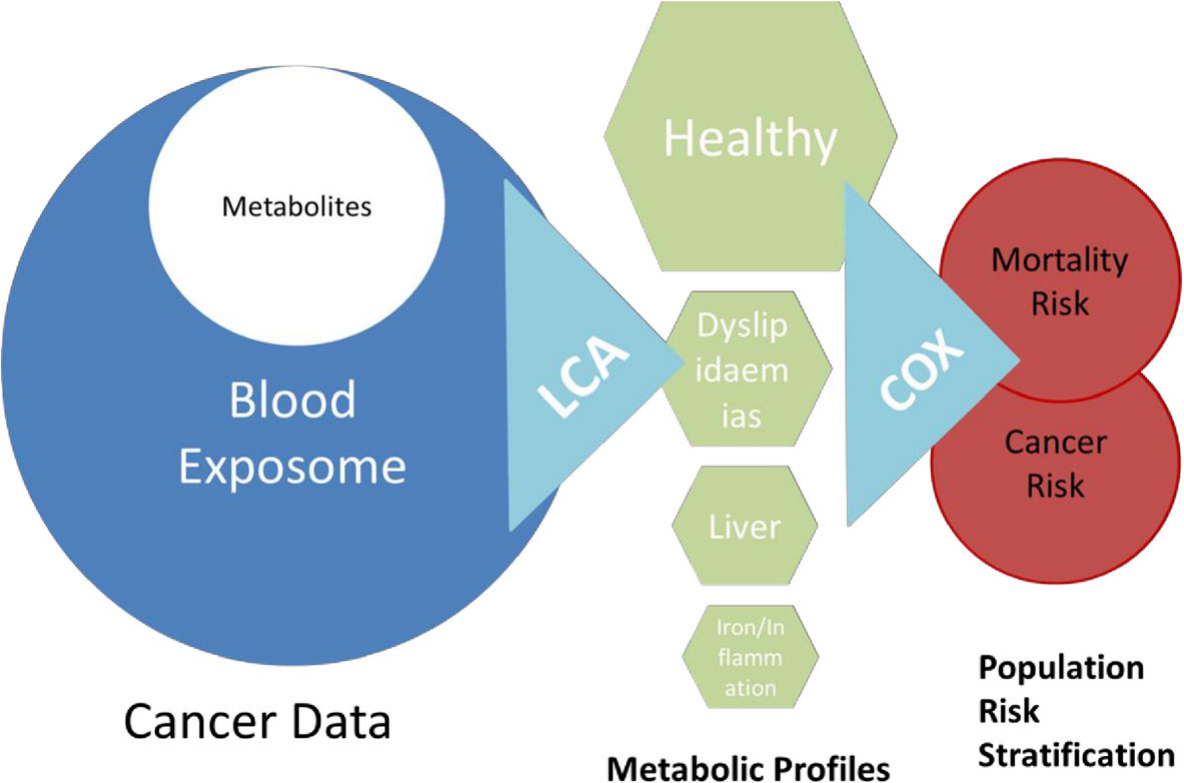
Study statistical pipeline describing the methodology followed in the project. We explored the blood exposome using metabolic markers of the population to assess how population heterogeneity is associated with cancer risk and mortality

The above-described three classes of abnormal metabolic profiles were all associated with an increased risk of cancer and worse survival, as compared to the healthy class. The findings therefore confirm the key importance of these metabolisms in the maintenance of the intracellular homeostasis and how their unbalance can be related with the etiology of cancer disease and mortality [2]. The LCA adapted in this study thus illustrates how a biomarker-wide approach can help assess markers of the blood exposome in the context of carcinogenesis and mortality [29] (Fig. 3).

More specifically, individuals presenting **abnormal liver function** markers carried worse outcomes in terms of overall cancer risk and cancer death, and a positive association with digestive, connective and endocrine cancers diagnosis. Moreover, the participants with this profile had a higher probability of overall death. These results are consistent with previous published data. A positive association between elevated GGT and overall cancer risk, with no interaction of ALT, was found in the AMORIS cohort previously [30], and it was also reported in other large cohort studies [31, 32]. These studies also found strong associations with elevated levels of GGT and digestive and respiratory cancer incidence. Elevated GGT has been associated with mortality from all causes, liver disease, cancer and diabetes, while ALT only showed associations with liver disease death in a large US cohort [33]. However, in a study based on an elderly population it was found that GGT was associated with increased cardiovascular disease mortality, and ALP and AST with increased cancer-related mortality [34]. Moreover, a meta-analysis evaluating the associations between liver enzymes and all-cause mortality found positive independent associations of baseline levels of GGT and ALP with all-cause mortality [35]. In the present study, the liver biomarker profile was positive associated with all the outcomes studied, suggesting a key role of this pathway in the development of cancer, probably related with its active role maintaining the intracellular redox regulation. Further investigations are necessary to establish the potential of the altered liver enzyme profile as a tool for cancer risk stratification.

Individuals allocated to the **lipid profile** presented positive associations with cancer mortality, and overall mortality and higher risk of lymphatic and hematopoietic cancers. The link between hyperlipidemia and mortality has been studied broadly, with associations with established links for cancer and all-cause mortality [36–38]. The association between lipids and lymphatic and hematopoietic cancers is more controversial, as other studies found an inverse association for these cancers and high levels of serum cholesterol [39, 40]. However, a systematic literature review from 2016 found no association [41].

Participants clustered in the **unbalanced iron profile and inflammation** had an increased risk of endocrine, buccal and oral cancers and were observed to have a higher risk of all-causes death. Altered inflammation and iron metabolisms are key metabolic ‘hallmarks of cancer’ [2, 42, 43]. Our observation of an association with an increased risk of buccal and oral cancer corroborates previous findings in AMORIS [42].

### Population heterogeneity and risk stratification: the need for data reduction techniques

The modulation effect of population heterogeneity on the association between potential risks factors and disease is a new avenue to understand the variability of risk in the population [44]. For instance, in a targeted metabolomics exercise Shan et al. performed a principal component analysis and time to event analysis identifying metabolic profiles to predict risk of CVD [13]. Another study used Monte Carlo Cross Validation and Lasso logistic regression to evaluate serum biomarkers as an alternative to fecal immunochemical testing to improve detection of colorectal cancer [11]. In 2010, the European Prospective Investigation on Cancer and Nutrition (EPIC) cohort reported that a specific prediagnostic plasma phospholipid fatty acid profile could predict the risk of gastric cancer [45]. As rationalized in the HELIX project, these multiple profiling approaches aim to identify groups of individuals in the population that share a similar exposome that might account for differences on the specific risk of study [46].Together with these studies, our systematic data integration approach based on LCA demonstrates the potential of investigating population heterogeneity using metabolic profiling as risk factors for long term cancer risk and mortality prediction. However, in order to establish the prediction capability of these LCA metabolic profiles and implement their use in a clinical setting, further studies to validate the results whilst allowing to measure sensitivity and specificity, will need to be conducted such as a nested case-control in AMORIS that could determine the predictive capabilities of the metabolic profiles to estimate cancer risk and mortality.

## Strengths and limitations

The present study has been conducted in a large and well-defined population, applying a multi-faced approach covering main biological pathways to assess biomarker profiles that could indicate cancer risk, cancer survival and mortality. The major strength of these analyses lies in the innovative avenue to study population heterogeneity and susceptibility to disease and mortality in a large cohort of participants with multiple measurements, all measured on fresh blood samples on the same day at the same clinical laboratory. We included all the markers available in the cohort for a large population (*n* > 13000), however not every marker of the central metabolic pathways was available in the database (i.e. Complete Blood Count). Life-style factors established as cancer risk factors such as tobacco smoking, low physical activity, poor diet, alcohol intake, obesity and hypertension were partially available in AMORIS which limited their used in the study. To mitigate the lack of some of these external factors such as BMI, the analyses have been adjusted for Charlson Comorbidity Index which includes comorbidities such as obesity and hypertension. The lack of others life-style factors such as alcohol consumption was mitigated by using information on serum biomarkers such as gamma glutamyl transferase and other liver enzymes. All participants were selected by analyzing blood samples from health check-ups in non-hospitalized individuals from the greater Stockholm area ensuring good internal validity in the study. Future studies will benefit from a longitudinal approach with repeated serum markers measurements that will capture the population phenotypic variations in relation to disease over long periods of time and will help to improve our understanding of the biomarkers’ impact on carcinogenesis and mortality.

## Conclusion

Our findings support the recently expressed need for a shift from the classical epidemiological approach of assessing one exposure to a systemic approach with multiple exposures. The LCA adapted in this study illustrates how a biomarker-wide approach can help assess population susceptibility to disease and provide insight into disease etiology in the context of carcinogenesis and mortality (Fig. 3). Given the environmental and genetic modulation of metabolic molecules, metabolic profiling based on standard of care serum markers could become a useful non-invasive predictive signature for risk stratification and an important area of research for mechanisms and clinical relevance.

## Methods

### Study design and study population

The AMORIS study, a large prospective cohort study, has been described in detail elsewhere [19, 47, 48]. Briefly, the AMORIS database is based on linkages with the Central Automation Laboratory (CALAB) database, which analyzed fresh blood samples from subjects from the greater Stockholm area. All individuals were either healthy individuals referred for clinical laboratory testing as part of a general health check-up or outpatients between 1985 and 1996. The AMORIS cohort has been linked to several Swedish national registries such as the National Cancer Register, the Patient Register, the Cause of Death Register, the consecutive Swedish Censuses during 1970–1990, and the National Register of Emigration, using the Swedish 10-digit personal identity number. These linkages provide detail information on demographics, lifestyle, socio-economic status, vital status, cancer diagnosis, comorbidities and emigration. The AMORIS study conformed to the declaration of Helsinki and was approved by the ethics board of the Karolinska Institute.

From the AMORIS cohort, we included all individuals aged 20 years or older with measurements for the following serum biomarkers (*n* = 13,615), which were all measured on the same day, using fully automated methods with automatic calibration performed on fresh blood samples, at the same laboratory (CALAB) of high quality according to international blinded testing [49] (Additional file 1: Table S1 and Table S2): total cholesterol (TC) (mmol/L), triglycerides (TG) (mmol/L), apolipoprotein A-1 (ApoA-I) (g/L), apolipoprotein B (ApoB) (g/L), high density lipoprotein (HDL) (mmol/L), low density lipoprotein (LDL) (mmol/L), glucose (mmol/L), fructosamine (FAMN) (mmol/L), gamma-glutamyl transferase (GGT) (IU/L), alanine aminotransferase (ALT) (IU/L), aspartate aminotransferase (AST) (IU/L), albumin (g/L), leukocytes (WBC) (10^9^ cells/L), C-reactive protein (CRP) (mg/L), iron (FE) (μmol/L), total iron binding capacity (TIBC) (mg/dL), creatinine (μmol/L), phosphate (mmol/L) and calcium (mmol/L). All methods have previously been described [48].

These biomarkers were selected to reflect common metabolic pathways: lipid (TC, TG, ApoA-I, ApoB, HDL and LDL) and glucose metabolism (Glucose, FAMN), liver function (GGT, ALT and AST), inflammation (Albumin, WBC and CRP), iron metabolism (FE and TIBC), kidney function (Creatinine) and phosphate (Phosphate and Calcium). The blood metabolites included in the analysis were all the standard serum markers available from routine health check-ups. Most of the markers included have been previously studied individually in AMORIS, however no systemic integrative approach to examine the metabolic markers interactions and susceptibility to cancer has been conducted to date [30, 42, 50–59]. All participants were free from cancer at time of study entry and none were diagnosed with cancer within the first three years of follow-up to avoid reverse causation.

The main exposure variables for the analyses were the above-mentioned metabolic biomarkers, for which the values were categorized using standardized clinical cut-offs based on recognized medical criteria to facilitate interpretation of the results (Additional file 1: Table S2). The main outcomes were first cancer diagnosis, as registered in the National Cancer Register using ICD-9 for the years 1987–1992, ICD-O/2 for years 1993–2004 and for year 2005 onwards has been coded in ICD-O/3), and mortality. As secondary outcomes, we explored those cancer types for which there were more than 30 events during follow-up. Likewise, cancer mortality was explored. Follow-up time was assessed specifically for each of the outcomes studied. For cancer diagnosis, follow-up time was defined as time from blood drawn until date of first cancer diagnosis, death, emigration or study closing date (31st of December 2012), whichever occurred first. The follow-up time for death was described as time from blood drawn until date of death, emigration or study closing date (31st of December 2012), whichever occurred first.

Information on the following potential confounders was also incorporated: age, sex and comorbidities. The latter was quantified using the Charlson Comorbidity Index (CCI) calculated based on data from the National Patient Register. The CCI comprises 19 disease categories, all assigned a weight. The sum of an individual’s weights was used to create the CCI ranging from no comorbidity to severe comorbidity (0, 1, 2, and ≥ 3) [60].

### Data analysis

First, we calculated **Pearson correlation coefficients** to measure the strength of association between the biomarkers included in the analysis. Pearson’s correlation analyses showed strong correlation between the different biomarkers in the lipid metabolism (TC, LDL and ApoB (r > 0.7); HDL and ApoA-I (r > 0.8)). We replaced the individual lipid biomarkers by the established ApoB/ApoA-I ratio and log (TG/HDL) ratio [20, 49, 61, 62] to avoid collinearity and to comply with the principle of local independence as required by latent class analysis [63]. Most of the markers were normally distributed except from the liver biomarkers.

**Latent Class Analysis** (LCA) [63, 64] is a model-based clustering method that reduces the dimension of the data by clustering covariates into latent classes, using a probabilistic model that describes the data distribution, and it assesses the probability that individuals belong to certain latent classes. LCA avoids the use of a linear combination or a random distance definition to reduce the number of covariates [65] and has recently been employed in health sciences [21, 66]. More specifically, we applied LCA to characterize different classes of individuals based on their metabolic profiles [67] and to evaluate intrinsic associations between the biomarkers, using the poLCA package [68] in R statistical programming language. We first determined the optimal number of LCA-derived classes by executing step-wise models with different numbers of classes, starting with the null model and adding one extra class in each model until reaching the total number of biomarkers in the data, while the model kept converging into a local maximum likelihood. The criterions used for model selection (Akaike information criterion (AIC), Bayesian information criterion (BIC) and Chi-squared distribution) were evaluated to estimate the best goodness of fit model and to define the optimal number of LCA-derived metabolic classes that characterized our dataset. To identify which sets of biomarkers predominantly explained each latent class, how the classes were distributed across the study population and which individuals were allocated to each class, we assessed the conditional probabilities, mixed proportions and class memberships of the best fitted latent class model.

Once each subject was assigned to its LCA-derived metabolic class, we conducted **multivariable Cox proportional hazard regression** to examine whether the LCA-derived metabolic classes were associated with long term risk of overall cancer as well as specific cancer types. In addition, we evaluated how the classes were associated with all cause-death and cancer-specific death. All models were adjusted for age, sex, and CCI. We performed a sensitivity analysis using age as a time-scale, as this is potentially a strong confounder. Moreover, Schoenfeld residuals were tested to ensure the proportional hazard assumption of the multivariable cox proportional hazard regression analysis.

Data management and statistical analyses were performed using Statistical Analysis Systems (SAS) release 4.3 (SAS Institute, Cary, NC) and R version 3.0.2 (R Foundation for Statistical Computing, Vienna, Austria).

## Additional file

Additional file 1: **Table S1.** Laboratory fully automated methods with automatic calibration were performed at one accredited laboratory (CALAB to measure the serum biomarkers examine in the study. **Table S2.** Panel of serum markers describing standard medical cut-offs information. **Table S3.** Characteristics of the study population by LCA-derived metabolic classes. (DOCX 28 kb)

## Data Availability

The authors can confirm that for ethical and legal reasons imposed there are restrictions to the allowance of general public access to the data underlying the findings of this study. The database is formed of not only the AMORIS cohort but is a merged database. This includes AMORIS plus information from the Swedish National Patient Registry, the National Cause of Death Registry, SWEDEHEART, the Work Lipids, Fibrinogen study, the Cohort of Swedish Men Study, the Swedish Mammography Cohort, the cohort of 60-year-old subjects in Stockholm, the Sollentuna Primary Prevention study and the National Prescribed Drug Register. The merged database from these sources contain sensitive information and is therefore anonymized and located in a security server with restricted access at the institute of Environmental medicine, Karolinska Institutet in Stockholm. Professor Maria Feychting (maria.feychting@ki.se) and Sofia Carlsson (sofia.carlsson@ki.se) are both members of the Steering Committee of the AMORIS cohort and are based on the Unit of Epidemiology, Institute of Environmental Medicine hosting the database. They would both be able to respond to external requests for data access given that the interested party can obtain approval from the data owners including the National Board of Health and Welfare in Sweden (http://www.socialstyrelsen.se/english) and Statistics Sweden (http://www.scb.se/en_/) as well as from the owners of the research registers at Karolinska Institutet, Stockholm. Sweden. To ensure persistent and long-term database storage and availability, AMORIS cohort database is stored at the Institute of Environmental Medicine and the storage follows the principles kept at Karolinska Institutet. The database can be accessed after permission and considering the restrictions by remote access through a secure LAN solution.

## Abbreviations

AIC: Akaike information criterion
ALT: Alanine Aminotransferase
AMORIS: Apolipoprotein MOrtality RISk Study
ApoA-I: Apolipoprotein A-1
ApoB: Apolipoprotein B
AST: Aspartate Aminotransferase
BIC: Bayesian information criterion
CALAB: Central Automation Laboratory
CCI: Charlson Comorbidity Index
CRP: C-reactive protein
FAMN: Fructosamine
FE: Iron
GGT: Gamma-glutamyl Transferase
HDL: High Density Lipoprotein
ICD-9: International Classification of Diseases 9th Revision
ICD-O/2: International Classification of Diseases for Oncology 2nd Revision
ICD-O/3: International Classification of Diseases for Oncology 3rd Revision
LCA: Latent Class analysis
LDL: Low Density Lipoprotein
SAS: Statistical Analysis Systems
TC: Total Cholesterol
TG: Triglycerides
TIBC: Total Iron Binding Capacity
WBC: Leukocytes
WHO: World Health Organization
