# Supplementary material for: Metabolic profiles to predict long-term cancer and mortality: the use of latent class analysis
Source: BMC Mol Cell Biol. 2019 Jul 23;20:28. doi: 10.1186/s12860-019-0210-7 (PMC6651931; doi:10.1186/s12860-019-0210-7)
Supplement: Supplementary file 1 — Table S1. Laboratory fully automated methods with automatic calibration were performed at one accredited laboratory (CALAB to measure the serum biomarkers examine in the study. Table S2. Panel of serum markers describing standard medical cut-offs information. Table S3. Characteristics of the study population by LCA-derived metabolic classes. (DOCX 28 kb) [file 12860_2019_210_MOESM1_ESM.docx]

**Additional file 1**

**Table S1| Laboratory fully automated methods with automatic calibration were performed at one accredited laboratory (CALAB to measure the serum biomarkers examine in the study.**

|  | **Instrument** | **Method** | **Total imprecision ^a^** |
| --- | --- | --- | --- |
| **Total cholesterol** | Technicon DAX^TM^ 96 Multichannel Analyzer (Bayer Diagnostics, Tarrytown, USA) | CHOD-PAP: Enzymatic cholesterol assay based on cholesterol esterase and cholesterol oxidase conversion followed by a Trinder-type sequence of reactions | ≤2.7% CV |
| **Triglycerides** | Technicon DAX^TM^ 96 Multichannel Analyzer (Bayer Diagnostics, Tarrytown, USA) | GPO-PAP: Enzymatic determination of glycerol with glycerol-phosphate-oxidase (GPO) after hydrolysis with lipoprotein lipase | ≤5.0% CV |
| **Apolipoprotein A-I** | Technicon DAX^TM^ 96 Multichannel Analyzer (Bayer Diagnostics, Tarrytown, USA) | Immunoturbidimetry using polyclonal antisera from Orion (Helsinki, Finland) | <4.0% CV |
| **Apolipoprotein B** | Technicon DAX^TM^ 96 Multichannel Analyzer (Bayer Diagnostics, Tarrytown, USA) | Immunoturbidimetry using polyclonal antisera from Orion (Helsinki, Finland) | <4.0% CV |
| **Glucose** | Technicon DAX^TM^ 96 Multichannel Analyzer (Bayer Diagnostics, Tarrytown, USA) | GOD-PAP method: Enzymatic colorimetric test in which glucose in serum reacts with oxygen to give gluconate and hydrogen peroxide in the presence of glucose oxidase | <2.2% CV |
| **Gamma-glutamyl transferase** | Technicon DAX^TM^ 96 Multichannel Analyzer (Bayer Diagnostics, Tarrytown, USA) | Enzymatic colorimetric test using reagents from Randox Laboratories Ltd, Crumlin, UK | ≤6.0% CV |
| **Alanine aminotransferase** | Technicon DAX^TM^ 96 Multichannel Analyzer (Bayer Diagnostics, Tarrytown, USA) | Enzymatic UV-test according to International Federation for Clinical Chemistry (including incubation with pyridoxal phosphate) | ≤6.0% CV |
| **Albumin** | Technicon DAX^TM^ 96 Multichannel Analyzer (Bayer Diagnostics, Tarrytown, USA) | Bromocresol green method | ≤2.0% CV |
| **Leukocytes** | Coulter^R^ STKS Haematology System (Coulter Corporation, Hialeah, USA)  Includes a PC-based Management system | The STKS is a haematology flow cytometer that automatically performs blood cell counting from whole blood samples | <2.7% CV |
| **C-reactive protein** | Technicon DAX^TM^ 96 Multichannel Analyzer (Bayer Diagnostics, Tarrytown, USA) | Immunoturbidimetry using polyclonal antisera from Orion (Helsinki, Finland) | ≤12.0% CV |

a CV: Coefficient of Variation

**Table S2| Panel of serum markers describing standard medical cut-offs information.**

| **Biomarkers** | **Cut-off low** | **Cut-off high** | **Units** |
| --- | --- | --- | --- |
| **Total Cholesterol** | - | 6.50 | mmol/L |
| **Triglycerides** | - | 1.71 | mmol/L |
| **ApoA-1** | 1.05 | - | g/L |
| **ApoB** | - | 1.50 | g/L |
| **HDL** | 1.30 | - | mmol/L |
| **LDL** | - | 4.10 | mmol/L |
| **ApoB/ApoA-I** | - | 1.00 | a |
| **log (TG/HDL)** | - | 0.50 | a |
| **Glucose** | - | 6.10 | mmol/L |
| **Fructosamine** | - | 2.60 | mmol/L |
| **GGT** | 36.00 | 72.00 | IU/L |
| **ALT** | - | 50.00 | IU/L |
| **AST** | - | 45.00 | IU/L |
| **Albumin** | 35.00 | - | g/L |
| **WBC** | - | 10.00 | 10^9^cells/L |
| **CRP** | - | 10.00 | mg/L |
| **Iron** | 9.00 - 11.00 | 30.00 - 31.00 | µmol/L |
| **TIBC** | 0.25 | 0.38 | mg/dL |
| **Creatinine** | 45.00 - 60.00 | 90.00 - 100.00 | µmol/L |
| **Phosphate** | 0.70 - 0.80 | 1.40 | mmol/L |
| **Calcium** | 2.20 | 2.60 | mmol/L |

The following abbreviations have been used in table 2: Total Cholesterol (TC), Triglycerides (TG), Apolipoprotein A-1 (ApoA-1), Apolipoprotein B (ApoB), High Density Lipoprotein (HDL), Low Density Lipoprotein (LDL), Fructosamine (FAMN), Gamma-Glutamyl transferase (GGT), Alanine aminotransferase (ALT), Aspartate aminotransferase (AST), Leukocytes (WBC), C-reactive protein (CRP), Iron (FE) and Total iron binding capacity (TIBC).

a Ratios are dimensionless

**Table S3| Characteristics of the study population by LCA-derived metabolic classes.** All the serum markers are dichotomized using the standardized clinical cut-offs. Clinically abnormal cut-off values are highlighted for each biomarker.

|  | **Class 1**  **Normal**  **N=8,612 (63.25%)** | **Class 2**  **Lipids**  **N=2,936 (21.56%)** | **Class 3**  **Liver**  **N=1,262 (9.27%)** | **Class 4**  **Inflammation/Iron N=805 (5.91%)** |
| --- | --- | --- | --- | --- |
| **Age (years)** |  |  |  |  |
| Mean(SD) | 51.25 (15.46) | 55.17 (13.04) | 48.98 (12.34) | 51.71 (15.21) |
| Under 40 | 2126 (24.69) | 360 (12.26) | 295 (23.38) | 170 (21.12) |
| 40-50 | 2192 (25.45) | 699 (23.81) | 415 (32.88) | 244 (30.31) |
| 50-60 | 1779 (20.66) | 813 (27.69) | 313 (24.80) | 160 (19.88) |
| Above 60 | 2515 (29.20) | 1064 (36.24) | 239 (18.94) | 231 (28.70) |
| **Sex** |  |  |  |  |
| Female | 5742 (66.67) | 1076 (36.65) | 320 (25.36) | 450 (55.90) |
| Male | 2870 (33.33) | 1860 (63.35) | 942 (74.64) | 355 (44.10) |
| **Socio-economics Status** | |  |  |  |
| High | 3959 (45.97) | 1515 (51.60) | 672 (53.25) | 347 (43.11) |
| Low | 3257 (37.82) | 972 (33.11) | 466 (36.93) | 312 (38.76) |
| Not employed or missing | 1396 (16.21) | 449 (15.29) | 124 (9.83) | 146 (18.14) |
| **Educational Status** |  |  |  |  |
| High | 2846 (34.91) | 840 (30.26) | 388 (32.17) | 239 (31.04) |
| Middle | 3525 (43.24) | 1156 (41.64) | 498 (41.29) | 316 (41.04) |
| Low | 1782 (21.86) | 780 (28.10) | 320 (26.53) | 215 (27.92) |
| Missing b | 459 (5.33) | 160 (5.45) | 56 (4.44) | 35(4.35) |
| **CCI** |  |  |  |  |
| 0 | 7959 (92.42) | 2497 (85.05) | 1113 (88.19) | 689 (85.59) |
| 1 | 495 (5.75) | 294 (10.01) | 103 (8.16) | 71 (8.82) |
| 2 | 101 (1.17) | 71 (0.52) | 24 (1.90) | 25 (3.11) |
| **3+** | 57 (0.66) | 74 (0.54) | 22 (1.74) | 20 (2.48) |
| **Total Cholesterol (mmol/L)** | |  |  |  |
| Mean(SD) | 5.62 (1.10) | 6.43 (1.13) | 5.99 (1.26) | 5.42 (1.14) |
| < 6.50 | 6753 (78.41) | 1530 (52.11) | 837 (66.32) | 654 (81.24) |
| **≥ 6.50** | 1859 (21.59) | 1406 (47.89) | 425 (33.68) | 151 (18.76) |
| **Triglycerides (mmol/L)** | |  |  |  |
| Mean(SD) | 1.06 (0.47) | 2.35 (1.25) | 1.99 (1.32) | 1.33 (0.84) |
| < 1.71 | 7843 (91.07) | 993 (33.82) | 653 (51.74) | 639 (79.38) |
| **≥ 1.71** | 769 (8.93) | 1943 (66.18) | 609 (48.26) | 166 (20.62) |
| **Apolipoprotein A-1 (g/L)** | |  |  |  |
| Mean(SD) | 1.48 (0.22) | 1.33 (0.20) | 1.38 (0.22) | 1.40 (0.22) |
| **< 1.05** | 88 (1.02) | 157 (5.35) | 52 (4.12) | 31 (3.85) |
| ≥ 1.05 | 8524 (98.98) | 2779 (94.65) | 1210 (95.88) | 774 (96.15) |
| **Apolipoprotein B (g/L)** | |  |  |  |
| Mean(SD) | 1.11 (0.29) | 1.52 (0.34) | 1.34 (0.38) | 1.14 (0.33) |
| < 1.50 | 7828 (90.90) | 1499 (51.06) | 875 (69.33) | 700 (86.96) |
| ≥ **1.50** | 784 (9.10) | 1437 (48.94) | 387 (30.67) | 105 (13.04) |
| **HDL Cholesterol (mmol/L)** | |  |  |  |
| Mean(SD) | 1.69 (0.36) | 1.18 (0.39) | 1.32 (0.42) | 1.50 (0.40) |
| **< 1.03** | 144 (1.67) | 950 (32.36) | 272 (21.55) | 91 (11.30) |
| ≥ 1.03 | 8468 (98.33) | 1986 (67.64) | 990 (78.45) | 714 (88.70) |
| **LDL Cholesterol (mmol/L)** | |  |  |  |
| Mean(SD) | 3.45 (0.98) | 4.21 (1.06) | 3.79 (1.09) | 3.38 (1.05) |
| < 4.10 | 6551(76.07) | 1362 (46.39) | 805 (63.79) | 627 (77.89) |
| ≥ **4.10** | 2061 (23.93) | 1574 (53.61) | 457 (36.21) | 178 (22.11) |
| **Glucose (mmol/L)** |  |  |  |  |
| Mean(SD) | 4.89 (0.75) | 5.96 (2.29) | 5.80 (2.18) | 5.26 (1.87) |
| < 6.11 | 8316 (96.56) | 2188 (74.52) | 989 (78.37) | 730 (90.68) |
| ≥ **6.11** | 296 (3.44) | 748 (25.48) | 273 (21.63) | 75 (9.32) |
| **Fructosamine (mmol/L)** | |  |  |  |
| Mean(SD) | 2.04 (0.18) | 2.22 (0.37) | 2.15 (0.34) | 2.05 (0.28) |
| < 2.6 | 8597 (99.83) | 2619 (89.20) | 1191 (94.37) | 777 (96.52) |
| ≥ **2.6** | 15 (0.17) | 317 (10.80) | 71 (5.63) | 28 (3.48) |
| **GGT (IU/L)** |  |  |  |  |
| Mean(SD) | 22.93 (23.71) | 38.18 (31.99) | 93.70 (117.85) | 30.24 (26.33) |
| Normal (<18) | 4713 (54.73) | 447 (15.22) | 33 (2.61) | 318 (39.50) |
| **Normal high (18-36)** | 2959 (34.36) | 1453 (49.49) | 284 (22.50) | 287 (35.65) |
| **Elevated (36-72)** | 731 (8.49) | 780 (26.57) | 442 (35.02) | 145 (18.01) |
| **Highly elevated (>72)** | 209 (2.43) | 256 (8.72) | 503 (39.86) | 55 (6.83) |
| **AST (IU/L)** |  |  |  |  |
| Mean(SD) | 20.05 (6.55) | 21.24 (6.49) | 46.41 (52.16) | 21.61 (20.46) |
| < 45 | 8565 (99.45) | 2925 (99.63) | 876 (69.41) | 789 (98.01) |
| **≥ 45** | 47 (0.55) | 11 (0.37) | 386 (30.59) | 16 (1.99) |
| **ALT (IU/L)** |  |  |  |  |
| Mean(SD) | 21.60 (9.35) | 28.32 (10.02) | 84.71 (91.11) | 23.59 (12.90) |
| < 50 | 8578 (99.61) | 2936 (100.00) | 0 (0.00) | 782 (97.14) |
| ≥ **50** | 34 (0.39) | 0 (0.00) | 1262 (100.00) | 23 (0.17) |
| **Albumin (g/L)** |  |  |  |  |
| Mean(SD) | 42.98 (2.71) | 43.14 (2.72) | 44.09 (3.01) | 41.79 (3.31) |
| **<35** | 6 (0.07) | 7 (0.24) | 3 (0.24) | 12 (1.49) |
| >35 | 8606 (99.93) | 2929 (99.76) | 1259 (99.76) | 793 (98.51) |
| **Leukocytes (10^9^ cells/L)** | |  |  |  |
| Mean(SD) | 6.24 (1.79) | 6.89 (1.99) | 6.55 (2.14) | 7.56 (2.64) |
| <10 | 8374 (97.24) | 2703 (92.06) | 1206 (95.56) | 673 (83.60) |
| ≥ **10** | 238 (2.76) | 233 (7.94) | 56 (4.44) | 132 (16.40) |
| **C-Reactive Protein (mg/L)** | |  |  |  |
| Mean(SD) | 4.99 (15.41) | 5.51 (12.17) | 5.78 (7.53) | 16.63 (24.12) |
| <10 | 7764 (90.15) | 2557 (87.09) | 1095 (86.77) | 442 (54.91) |
| **10-15** | 717 (8.33) | 265 (9.03) | 106 (8.40) | 108 (13.42) |
| **15-25** | 62 (0.72) | 78 (2.66) | 33 (2.61) | 92 (11.43) |
| **25-50** | 48 (0.56) | 31 (1.06) | 23 (1.82) | 98 (12.17) |
| **>50** | 21 (0.24) | 5 (0.17) | 5 (0.40) | 65 (8.07) |
| **Iron (µmol/L)** |  |  |  |  |
| Mean(SD) | 18.77 (5.43) | 17.99 (4.70) | 20.00 (6.65) | 8.86 (2.79) |
| **Low** | 1 (0.01) | 4 (0.14) | 36 (2.85) | 595 (73.91) |
| Normal | 8276 (96.10) | 2888 (98.37) | 1138 (90.17) | 210 (26.09) |
| **High** | 335 (3.89) | 44 (1.50) | 88 (6.97) | 0 (0.00) |
| **TIBC (mg/dL)** |  |  |  |  |
| Mean(SD) | 0.32 (0.10) | 0.30 (0.09) | 0.33 (0.12) | 0.15 (0.05) |
| **Low** | 2050 (23.80) | 877 (29.87) | 335 (26.55) | 805 (100.00) |
| Normal | 4490 (52.14) | 1603 (54.60) | 557 (44.14) | 0 (0.00) |
| **High** | 2072 (24.06) | 456 (15.53) | 370 (29.32) | 0 (0.00) |
| **Creatinine (µmol/L)** | |  |  |  |
| Mean(SD) | 77.42 (15.03) | 84.80 (17.99) | 83.17 (13.92) | 79.22 (18.58) |
| **Low** | 20 (0.23) | 11 (0.37) | 5 (0.40) | 4 (0.50) |
| Normal | 7863 (91.30) | 2412 (82.15) | 1108 (87.80) | 705 (87.58) |
| **High** | 729 (8.46) | 513 (17.47) | 149 (11.81) | 96 (11.93) |
| **Phosphate (mmol/L)** | |  |  |  |
| Mean(SD) | 1.07 (0.16) | 1.06 (0.18) | 1.07 (0.19) | 1.10 (0.19) |
| Low | 70 (0.81) | 7 (0.24) | 8 (0.63) | 10 (1.24) |
| Normal | 8160 (94.75) | 2750 (93.66) | 1185 (93.90) | 701 (87.08) |
| **High** | 382 (4.44) | 179 (6.10) | 69 (5.47) | 94 (11.68) |
| **Calcium (mmol/L)** |  |  |  |  |
| Mean(SD) | 2.37 (0.09) | 2.39 (0.10) | 2.40 (0.10) | 2.35 (0.10) |
| Low | 110 (1.28) | 26 (0.89) | 13 (1.03) | 42 (5.22) |
| Normal | 8394 (97.47) | 2838 (96.66) | 1212 (96.04) | 751 (93.29) |
| **High** | 108 (1.25) | 72 (2.45) | 37 (2.93) | 12 (1.49) |
| **Log (triglycerides/HDL) c** | |  |  |  |
| mean(SD) | (-)0.54 (0.53) | 0.63 (0.79) | 0.29 (0.87) | (-)0.23 (0.75) |
| < 0.5 | 8576 (99.58) | 1166 (39.71) | 770 (61.01) | 685 (85.09) |
| **≥ 0.5** | 36 (0.42) | 1770 (60.29) | 492 (38.99) | 120 (14.91) |
| **ApoB/ApoA-I c** |  |  |  |  |
| mean(SD) | 0.76 (0.22) | 1.15 (0.28) | 0.99 (0.31) | 0.83 (0.28) |
| < 1.00 | 7582 (88.04) | 699 (23.81) | 683 (54.12) | 620 (77.02) |
| **≥ 1.00** | 1030 (11.96) | 2237 (76.19) | 579 (45.88) | 185 (22.98) |
| **Life Status** |  |  |  |  |
| Alive | 6815 (79.13) | 2066 (70.37) | 973 (77.10) | 603 (74.91) |
| Death | 1797 (20.87) | 870 (29.63) | 289 (22.90) | 202 (25.09) |
| Cancer | 1148 (13.33) | 490 (16.69) | 197 (15.61) | 121 (15.03) |

The following abbreviations have been used in Table 1: High Density Lipoprotein (HDL), Low Density Lipoprotein (LDL), Gamma-Glutamyl transferase (GGT), Alanine aminotransferase (ALT), Aspartate aminotransferase (AST) and Total iron binding capacity (TIBC).

a Clinically abnormal cut-off values are highlighted for each biomarker.

b The missing values are not included in the percentage of the Educational Status categories

c Ratios are dimensionless

*Clinical cut-offs

The following cut-offs criteria was applied:

**GGT reference interval:**

Low [GGT < 36 IU/L]

Normal [36 IU/L ≥ GGT <72 IU/L]

High [GGT ≥ 72 IU/L]

**Iron reference interval:**

Men [Low ≤ 11, Normal = 11-31, High ≥ 31]

Women [Low ≤ 9, Normal = 9-30, High≥ 30]

**TIBC reference interval:**

Men [Low ≤ 0.257, Normal = 0.257-0.379, High ≥ 0.379]

Women [Low ≤ 0.246, Normal = 0.246- 0.391, High ≥ 0.391]

**Creatinine reference interval:**

Men [Low ≤ 60, Normal = 60-100, High ≥ 100]

Women [Low ≤ 45, Norma l= 45-90, High ≥ 90]

**Phosphate reference interval:**

Men [Low ≤ 0.7, Normal = 0.7-1.4, High ≥ 1.4]

Women [Low ≤ 0.8, Normal = 0.8-1.4, High ≥1.4]

**Calcium reference interval per gender by age:**

Men

[Age < 40, Low ≤ 2.22, Normal = 2.22-2.60, High ≥2.60]

[Age 40-60, Low ≤ 2.20, Normal = 2.20 -2.59, High ≥2.59]

[Age > 60, Low ≤ 2.19, Normal= 2.19 -2.58, High ≥ 2.58]

Women

[Age < 40, Low ≤ 2.17, Normal = 2.17-2.56, High ≥2.56]

[Age 40-60, Low ≤2.19, Normal = 2.19-2.60, High ≥2.60]

[Age > 60, Low ≤ 2.21, Normal = 2.21-2.60, High ≥2.60]
